# Primary ovarian Burkitt’s lymphoma: a rare oncological problem in gynaecology: a review of literature

**DOI:** 10.1007/s00404-017-4478-6

**Published:** 2017-08-02

**Authors:** Anna Stepniak, Piotr Czuczwar, Piotr Szkodziak, Ewa Wozniakowska, Slawomir Wozniak, Tomasz Paszkowski

**Affiliations:** 3rd Department of Gynecology, Medical University of Lublin, ul. Jaczewskiego 8, 20-954 Lublin, Poland

**Keywords:** Burkitt’s lymphoma, Primary ovarian Burkitt’s lymphoma, Ovarian tumour, Non-Hodgkin lymphoma

## Abstract

**Purpose:**

This review presents the information about epidemiology, clinical manifestation, diagnosis and treatment of primary ovarian Burkitt’s lymphoma (BL), including a literature search of available BL cases. The purpose of this review is to draw clinicians’ attention to the possibility of ovarian BL occurrence, which may be important in the differential diagnosis of ovarian tumours.

**Methods:**

PubMed and Web of Science databases were searched using the keywords ‘‘Burkitt’s’’, ‘‘Lymphoma’’, ‘‘Ovarian’’, ‘‘Primary’’, ‘‘Burkitt’s lymphoma’’. Only cases with histopathologically confirmed diagnosis of primary ovarian BL were included in this review.

**Results:**

Fifty articles, reporting cases with an ovarian manifestation of primary non-Hodgkin’s lymphoma, were found. Twenty-one cases with a histopathologically confirmed BL were evaluated to compare various manifestations, treatment and prognosis in ovarian BL.

**Conclusions:**

Primary ovarian BL is a rare condition, included in the entity of non-Hodgkin lymphoma. The tumour can occur uni- or bilaterally in the ovaries with major symptoms such as abdominal pain or a large abdominal mass. Differential diagnosis, based on imaging features and pathological examination of the specimens, is essential for further treatment due to various aetiology of ovarian tumours. Although most of the patients suffering from ovarian BL underwent surgery after the ovarian tumour had been detected, surgical treatment is not the treatment of choice in patients with ovarian lymphoma. The mainstay of therapy is chemotherapy without further surgery. The prognosis is better if the chemotherapy protocol is more aggressive and followed by prophylactic central nervous system chemotherapy. Nowadays, multiagent protocols are administered, which improves the survival rate.

## Introduction

Burkitt’s lymphoma (BL) was first described by a British surgeon Denis Parsons Burkitt in Africa. It is a malignant lymphoma associated with the translocation between c-myc gene and immunoglobulin heavy locus, with the most common variant t(8;14)(q24;q32). BL is more common in children, while in adults, large cell lymphomas and low-grade B cell lymphomas predominate [1]. The 2016 revision of the World Health Organization classification of lymphoid neoplasms sustained the BL subtypes from 2008, which distinguished three types of this neoplasm: endemic, sporadic and immunodeficiency-associated [2]. BL, especially its endemic variant, is highly connected with Epstein-Barr virus infection, moreover, it is diagnosed more often in endemic malaria regions [3]. Sporadic BL subtype is diagnosed mostly in young adults (in the second and third decades) in Western Europe and North America. Interestingly, sporadic BL frequently shows extranodal manifestation, usually in the abdominal cavity [4]. Immunodeficiency-associated BL is mostly detected in HIV-positive patients, but may also be seen in patients after organ transplantations or in ones with primary immunodeficiency [4]. Knowles claims that BL occurs more often in HIV-positive individuals in comparison to the general population [5]. The main risk factors for BL include age, sex, geography and viral infections [6].

Aggressive lymphomas are rare findings in the ovaries. Ovarian lymphoma is usually the secondary manifestation of disseminated disease and uncommonly the primary extranodal manifestation. The statement “primary” and “secondary” involvement might be misleading due to the fact that ovarian lymphoma represents the extranodal manifestation of a systemic disease—disseminated BL. However, in the literature those descriptions are introduced for a clearer distinction of clinical course and outcome. Primary ovarian lymphoma (POL) is defined by the lack of other manifestations of the disease. It was suggested, that the origin of ovarian lymphomas is associated with the presence of pre-existing lymphoid tissue in the ovary [7]. Even though lymphocytes are in general absent in the ovaries, those surrounding the blood vessels at the hilum or those related to the corpus luteum are thought to be the cells of the tumour origin [8]. On the other hand, some authors claim that reactive lymphocytes may secondarily involve the ovary in response to inflammation (pelvic inflammatory disease, endometriosis) or autoimmune diseases and then may undergo a malignant transformation and arise as POL. The secondary involvement can be divided into two types: (a) an early expression of unknown extraovarian condition; (b) secondary ovarian involvement of disseminated systemic BL [9]. It is thought that there are four main pathways for BL cells to reach the ovaries: haematogenous, lymphatic, transcoelomic and direct [10]. However, the origin and pathogenesis of BL remain poorly understood and the abovementioned theories should be considered speculative.

POL accounts for 0.5% of non-Hodgkin lymphoma (NHL) and constitutes 1.5% of all ovarian neoplasms [9]. Differential diagnosis between the primary and secondary origin of ovarian BL may be challenging. Fox et al. presented the diagnostic criteria for POL in 1988:

1. The lymphoma should be confined to the ovary or the adjacent lymph nodes or structures at diagnosis;
2. There is no evidence of the disease in blood or bone marrow;
3. Remote involvement should appear at least a few months after ovarian involvement [11]. Paladugu et al. have suggested that at least 60 months should pass between diagnoses [12].

Survival rate and progression of the disease vary between primary and secondary origin, with more favourable outcomes in primary BL [13]. Better survival rates probably result from the fact that primary BL represents an early stage of the disease. That is why differential diagnosis is important mainly for prognosis, and not for the treatment decision. Nowadays the risk-adapted approach is used in selecting the treatment for most patients. Patients are classified as low- and high-risk individuals. The criteria for low-risk patients are as follows: non-bulky disease (<10 cm), early stage (I or II) disease, good performance status, and a normal lactate dehydrogenase (LDH) level. High-risk patients include those with higher staging, bone marrow infiltration, cerebral manifestation, elevated LDH level or tumour masses greater than 10 cm [14].

Not only BL can be localized in the ovary, but also other types of lymphomas such as diffuse large-cell, follicular lymphoma and sporadically precursor B-cell lymphoblastic lymphoma. Moreover, tumours of other origin e.g. metastatic carcinoma, small-cell carcinoma, adult granulosa cell tumour or dysgerminoma, may appear in the ovary and mimic BL [15]. Immunohistochemical studies enable to distinguish the type of the tumour and choose the best treatment regimen. Primary ovarian BL is a rare condition, therefore the literature data in this topic remains limited. The purpose of this review is to summarize the current knowledge regarding primary ovarian BL.

## Materials and methods

An extensive literature search was performed to collect data on primary ovarian BL. Relevant literature published between 1980 and 2016 was obtained from the PubMed and Web of Science databases. Additional papers were identified using the “related articles” button in PubMed. The following keywords were used: “Burkitt’s”, “Lymphoma”, “Ovarian”, “Primary”, “Burkitt’s lymphoma”. The search and selection of the literature was restricted to publications written in English. Only cases with histopathologically confirmed diagnosis of primary ovarian BL were included in this review.

## Results

### General results

A total of 50 articles, reporting cases with an ovarian manifestation of primary non-Hodgkin’s lymphoma, that were potentially relevant were identified. In 21 of the reported cases a histopathologically confirmed diagnosis of primary ovarian BL was made and these cases underwent a detailed evaluation. Our results show that there is no specific patient profile for ovarian BL manifestation. Surprisingly, the age of patients ranges widely, from 6 to 62 years-old. Although BL appears more frequently in children and young adults, the mean age in revised cases amounted 27.6 years-old. The reported symptoms, laboratory tests, imaging findings, histopathological findings, staging, treatment and prognosis in primary ovarian BL are discussed below. A detailed summary of these cases is presented in Table 1.

**Table 1:** Reported cases of primary ovarian Burkitt’s lymphoma

Case number	1st author	Age	Symptoms	Stage	Ovarian involvement	Treatment	Follow-up	References
1	Baloglu	24	Secondary amenorrhea, ascites, pleural effusion	IV Ann Arbor	Bilateral	Chemotherapy: Cyclophosphamide, Adriamycin, Vincristine, L-asparaginase, Prednisolone, plus intrathecal Methotrexate	REMISSION; autologous bone marrow transplantation; death 35 days after transplantation	[32]
2	Vang	62	Constitutional symptoms	I Ann Arbor	Unilateral	Chemotherapy, radiotherapy	Remission	[13]
3	Linden	24	Abdominal pain	II Ann Arbor	Unilateral	Surgery, chemotherapy	NA	[33]
4	Liang	38	Abdominal pain	I Ann Arbor	Unilateral	Chemotherapy, radiotherapy	Disease-free survival (0.5 year)	[34]
5	Mitra	23	Abdominal pain	I Ann Arbor	Unilateral	Radiotherapy	Disease-free survival (0.5 year)	[35]
6	Miyazaki	16	Abdominal pain, constipation	NA	Unilateral	Surgery, chemotherapy	Died after 171 days	[30]
7	Cyriac	13	Abdominal pain, fever	IV Ann Arbor/1 FIGO	Bilateral	Chemotherapy (LMB 89 protocol)	Disease-free survival (0.5 year)	[36]
8	Crawshaw	28	Abdominal pain, pregnancy	IV Ann Arbor/1 FIGO	Bilateral	Chemotherapy	NA	[7]
9	Chishima	25	Abdominal pain	IV Ann Arbor/1 FIGO	Bilateral	Surgery, chemotherapy (CHOP)	Disease-free survival (2.5 years)	[37]
10	Monterroso	21	Abdominal pain	NA	NA	Surgery, chemotherapy	Died after a year	[38]
11	Ng	20	Abdominal pain	1B FIGO	Bilateral	Surgery, chemotherapy (BFM regime), plus prophylactic intrathecal Methotrexate	Remission	[39]
12	Shacham-Abulafia	39	Night sweats, abdominal pain, dyspnea	IV Ann Arbor, 3 FIGO	Bilateral	Chemotherapy (R-Hyper-CVAD plus GMALL-B-ALL/NHL 2002)	Disease-free survival (0.5 year)	[17]
13	Bianchi	57	Neurological symptoms	IV Ann Arbor	Bilateral	Surgery, chemotherapy (intensive G-mall protocol)	NA	[40]
14	Danby	11	Abdominal pain, nausea, vomiting, anorexia, and weight loss	NA	Unilateral	Surgery, chemotherapy (Rituximab, Methotrexate, Cytarabine, intrathecal Hydrocortisone, Cytosine arabinoside, Methotrexate and Cyclophosphamide)	NA	[41]
15	Etonyeaku	18	Abdominal pain, lower abdominal swelling	NA	Bilateral	Surgery, chemotherapy (Cyclophosphamide, Vincristine, Methotrexate)	Died after second chemotherapy	[3]
16	Gottwald	27	Abdominal pain, ascites	NA	Bilateral	Surgery, chemotherapy (COP followed by CODOX-M + IVAC)	Disease-free survival (3 years)	[42]
17	Gutierrez	34	NA	IV Ann Arbor, 3 FIGO	Bilateral	NA	NA	[43]
18	Hatami	58	Abdominal mass	IV Ann Arbor	Bilateral	Surgery, chemotherapy (Vincristine, Rituximab, Methotrexate with Leucovorin and intrathecal Methotrexate)	Disease-free survival (3.5 years)	[21]
19	Munoz	30	Abdominal pain	NA	Bilateral	Surgery, chemotherapy (CODOX-M-IVAC plus Rituximab)	NA	[44]
20	Khan	6	Abdominal pain and masses	IV Ann Arbor	Bilateral	Surgery, chemotherapy (MCP-842 protocol)	NA	[45]
21	Mondal	6	Abdominal pain, difficulties with walking	II R Murphy staging	Bilateral	Surgery, chemotherapy (Magrath protocol using CODOX-M regimen)	Remission	[8]

*NA* not available

### Symptoms

BL is a rapidly progressive disease with an aggressive clinical course. Ovarian BL is mainly the manifestation of the sporadic BL subtype, which often presents as an abdominal tumour. The central nervous system (CNS) and bone marrow are frequently involved. According to the literature search in many cases a pelvic or abdominal mass was found incidentally during an ultrasound examination. Symptoms reported in the majority of the patients were similar, with abdominal pain as the main complaint (Table 1). Other signs and symptoms revealed during the diagnostic process were: complex adnexal or abdominal mass, pelvic pain and discomfort, abnormal vaginal bleeding, irregular menses, urination, bowel obstruction, ascites. If the CNS is involved, meningeal infiltration, headaches, visual impartment and paraplegia may occur [14]. Constitutional symptoms such as fever, nights sweats, fatigue and weight loss may also be seen.

### Laboratory tests

**Fig. 1:**
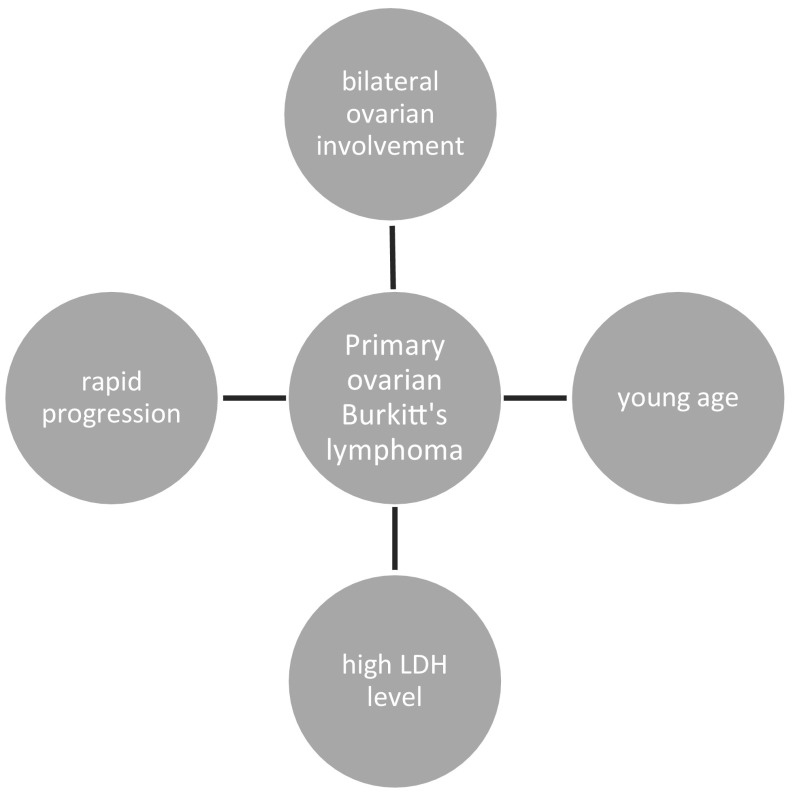
Factors suggestive of primary ovarian Burkitt’s lymphoma

Clinical examination followed by laboratory tests is the first step in the diagnostic process of BL. Complete blood cell count, coagulation studies, electrolytes, liver and renal function tests should be performed in all patients. Laboratory tests should also include serum levels of LDH and Ca-125, which were elevated in most of the reported cases (Fig. 1). Serum levels of CEA, Ca 19-9, AFP, β-HCG were within normal levels in patients with ovarian BL, and may be useful in the differential diagnosis of ovarian tumours as they are specific for other neoplasms [16]. Elevated serum levels of LDH were detected in most cases of BL, up to very high values such as 14,497 IU/ml with an average increase to approximately 2000 IU/ml [17]. Laboratory tests for HIV and EBV viral infections are also recommended. Additionally, in some cases evaluation of the cerebrospinal fluid, bone marrow aspiration and positron emission tomography may be useful in clarifying the diagnosis.

### Imaging findings

CT scan, MRI and ultrasound examination are useful in detecting abnormal masses in pelvic and/or abdominal cavities. Ferrozzi et al. describe the most typical imaging features of ovarian lymphoma on CT scan, ultrasound and MRI. CT scan shows hypodense lesions with mild contrast enhancement, whereas ultrasound presents a nonspecific appearance with hypoechogenic, homogeneous structures. MRI depicts homogeneous masses with hypointense T1-weighted images and slightly hyperintense T2-weighted images. The authors emphasize the importance of including ovarian lymphoma in the differential diagnosis of ovarian tumours. Interestingly, in 67% of reported cases of primary ovarian BL bilateral ovarian involvement was detected (Table 1). If ascites is absent and a homogeneous bilateral tumour appears in the ovaries the most likely diagnosis is an ovarian lymphoma [18]. Factors suggestive of primary ovarian Burkitt’s lymphoma are summarized on Fig. 1.

### Histopathological findings

The diagnosis of BL is made by obtaining tumour cells for histopathology, immunochemistry and flow cytometry. In most cases the revealed masses are surgically removed, the pathological evaluation of obtained specimens is essential for further recognition. Peritoneal cytology may be positive for malignant cells. A typical morphological spectrum shows high expression of B-cell markers: CD 20, CD 22, CD 19 and CD 10, as well as negative stain against cytokeratin and T-cell markers. Rosenwald claims that diagnosis of classical BL is based on the presence of a monotonous infiltrate of medium-sized blastic lymphoid cells that show round nuclei with clumped chromatin and multiple, centrally located nucleoli [19]. The microscopic view is mostly reminiscent of a starry-sky pattern, with a high proliferation rate and intermingled macrophages containing apoptotic debris, typical for BL. According to Haralambieva et al. the following criteria for immunophenotype during BL diagnosis should be met:

1. Ki-67 at proliferation rate around 90%,
2. breakpoints in Myc gene,
3. CD10 positive,
4. absence of Bcl-2 expression [20].

MYC gene rearrangements are typical for BL [21], but according to the WHO these translocations are not obligatory to diagnose BL [22].

### Staging

Staging plays an important role in the evaluation of the spread of the disease and allows the use of a uniform treatment approach. Additionally, it allows comparing treatment regimens and outcomes among various clinical units [23]. For ovarian BL there are multiple staging options, based on the tumour location, patient age and finally BL subtype. Ann Arbor classification is commonly used in adult patients with BL, while St. Jude/Murphy in paediatric patients. The Ann Arbor and St. Jude/Murphy classifications consist of four stages:

Stage I: single tumour (extranodal) or a single anatomic area (nodal).

Stage II: single extranodal tumour with regional node involvement, two single extranodal tumours on the same side of the diaphragm, primary gastrointestinal tumour, two or more nodal areas on the same side of the diaphragm.

Stage IIR: completely resected intra-abdominal disease.

Stage III: Two single extranodal tumours on opposite sides of the diaphragm, all primary intrathoracic tumours, all paraspinal or epidural tumours, all extensive intra-abdominal disease, two or more nodal areas on opposite sides of the diaphragm.

Stage IV: any of the above, with initial CNS and/or bone marrow involvement [4].

According to our literature search most of the patients with primary ovarian BL had advanced stages of the disease (IV Ann Arbor).

### Treatment

BL is an aggressive B-cell non-Hodgkin lymphoma with a very short doubling time and for this reason intensive multiagent chemotherapy regimens are considered to be the most appropriate therapeutic option for patients with BL [24]. Surgical treatment is not the treatment of choice in patients with ovarian lymphoma. However, surgical intervention plays an important role in the diagnostic process providing clinical information, staging and immunohistological examination. Despite its usefulness in the diagnostic process, surgical treatment should not delay the initiation of chemotherapy. Lu et al. claim that ovarian BL patients should undergo systemic evaluation before surgery, including ovarian fine-needle aspiration, bone marrow biopsy followed by cytology and flow cytometry from cavity effusions [1]. Therefore, according to Shacham et al. it is still unclear whether surgery should be incorporated for debulking in ovarian BL in view of the effectiveness of chemotherapy and the relatively high rate of mortality and morbidity associated with surgical interventions [17]. On the other hand, surgery may be indicated in case of acute abdominal complications of BL [25].

Our literature search revealed that 60% of the patients suffering from primary ovarian BL underwent surgery after the ovarian tumour had been detected (Table 1). After immunohistochemical examination and final diagnosis, the chemotherapy was introduced in 90% of the reported cases (in one case only radiotherapy was implemented and in one case there was no information about the treatment regimen used). Ovarian manifestation of BL is extremely sensitive to the chemotherapy similarly as other locations of BL and chemotherapy is the treatment of choice after final recognition. Bilateral ovarian removal in young patients seems to be more harmful than beneficial, but intensive chemotherapy may also lead to infertility in those patients.

Over the past 20 years chemotherapy regimens evaluated from less intensive protocols to highly aggressive multi-agent therapy. There is no worldwide standard of care for BL. Most of the adult BL treatment regimens have been adopted from paediatric protocols. Although intensive chemotherapy improved the outcomes in paediatric BL, in most trials increased age was independently associated with inferior outcomes [4].

The most frequently used protocols in adult patients are listed below:

1. The German Multicenter Study Group for Adult ALL (GMALL) developed two protocols: B-NHL 83 and B-NHL 86. The B-NHL 83 protocol consists of cyclophosphamide, prednisone, methotrexate, teniposide, cytarabine, doxorubicin, and leucovorin, in six 5-day cycles. The B-NHL 86 protocol consists of similar substances but replaces cyclophosphamide with ifosfamide and prednisone with dexamethasone [26].
2. The French LMB protocol starts with a cytoreductive COP regimen (low dose of cyclophosphamide, vincristine, and prednisone), followed by two induction COPADM cycles—high dose of methotrexate, cyclophosphamide, vincristine, doxorubicin, and prednisone [4].
3. The CODOX-M/IVAC protocol, developed by Magrath, consists of cyclophosphamide, vincristine, doxorubicin, high-dose methotrexate plus intrathecal therapy, alternated with ifosfamide, etoposide, high-dose cytarabine plus intrathecal therapy [7].
4. HyperCVAD protocol with or without rutiximab consisting of hyperfractionated cyclophosphamide, vincristine, doxorubicin, dexamethasone, and CNS prophylaxis [27].

The National Comprehensive Cancer Network panel recommends using CODOX-M or HyperCVAD for the low-risk patient with BL, and CODOX-M/IVAC with or without rituximab, or HyperCVAD alternating with methotrexate plus cytarabine, with or without rituximab for the high-risk patient [28].

### CNS prophylaxis

Triple intrathecal prophylaxis with methotrexate, cytarabine and corticosteroids was also used in some reported cases of ovarian BL, administered as a part of each of the treatment courses [22]. Prophylactic central nervous system treatment was implemented into the therapeutic regimen due to novel studies on the progression of the tumour, which indicated high propensity to spread to the CNS [23]. Intrathecal chemotherapy with methotrexate, prednisolone or cytosine arabinoside is recommended.

### Prognosis

Although BL has a high growth rate, the survival rate in paediatric patients is estimated at approximately 80%, whereas in adults the reported values are lower, but still very optimistic [20]. Aggressive therapies in young patients with BL have an excellent outcome, while treatment-related toxicity remains a big challenge in BL therapy, especially in older adults [29]. Five-year survival rate in advanced-stage disease has been estimated at 60–85%, however few data are available about the prognosis of BL with ovarian involvement [30]. The prognosis of patient’s outcome based on the International Prognostic Index (IPI) of non-Hodgkin’s lymphoma that includes Ann Arbor staging, patient age, serum lactate dehydrogenase (LDH) level, performance status and number of extranodal sites of lymphoma [31]. The prognosis is better if the chemotherapy protocol is more aggressive and followed by prophylactic central nervous system chemotherapy. Nowadays, multiagent protocols are administered, which improves the survival rate [21].

## Conclusion

Despite the rarity of primary ovarian BL, it should be taken into consideration in patients with abdominal circumference enlargement, pain localized in the abdominal cavity or pelvis and solid tumours in imaging studies. The importance of early recognition remains a key point in further therapy regarding the different treatment protocols for ovarian tumours of other origin. Most authors affirm that factors such as young age and bilateral manifestation of the ovarian tumour followed by rapid progression of the ovarian mass, should urge to be vigilant and to introduce the extended diagnostic procedure, which may confirm primary ovarian Burkitt’s lymphoma.
